# Characteristics of an Environmentally Monitored Prolonged Type 2 Vaccine Derived Poliovirus Shedding Episode that Stopped without Intervention

**DOI:** 10.1371/journal.pone.0066849

**Published:** 2013-07-31

**Authors:** Tapani Hovi, Anja Paananen, Soile Blomqvist, Carita Savolainen-Kopra, Haider Al-Hello, Teemu Smura, Hiroyuki Shimizu, Katarina Nadova, Zdenka Sobotova, Eugene Gavrilin, Merja Roivainen

**Affiliations:** 1 Virology Unit, Department of Infectious Disease Surveillance and Control, National Institute for Health and Welfare, a WHO Collaborating Centre for Poliovirus Surveillance and Enterovirus Research, Helsinki, Finland; 2 Department of Virology II, National Institute of Infectious Diseases, Tokyo, Japan; 3 Health Centre, Municipality of Skalica, Skalica, Slovak Republic; 4 National Reference Centre for Poliomyelitis, Authority of Public Health of the Slovak Republic, Bratislava, Slovak Republic; 5 Division of Communicable Diseases, Health Security and Environment, World Health Organization, Regional Office for Europe, Copenhagen, Denmark; Duke-NUS Gradute Medical School, Singapore

## Abstract

Vaccine derived poliovirus (VDPV) type 2 strains strongly divergent from the corresponding vaccine strain, Sabin 2, were repeatedly isolated from sewage in Slovakia over a period of 22 months in 2003–2005. Cell cultures of stool specimens from known immune deficient patients and from an identified putative source population of 500 people failed to identify the potential excretor(s) of the virus. The occurrence of VDPV in sewage stopped without any intervention. No paralytic cases were reported in Slovakia during the episode. According to a GenBank search and similarity plotting-analysis, the closest known relative of the first isolate PV2/03/SVK/E783 through all main sections of the genome was the type 2 poliovirus Sabin strain, with nucleotide identities in 5′UTR, P1, P2, P3, and 3′UTR parts of the genome of 88.6, 85.9, 87.3, 88.5, and 94.0 percent, respectively. Phenotypic properties of selected Slovakian aVDPV strains resembled those of VDPV strains isolated from immune deficient individuals with prolonged PV infection (iVDPV), including antigenic changes and moderate neurovirulence in the transgenic mouse model. One hundred and two unique VP1 coding sequences were determined from VDPV strains isolated from 34 sewage specimens. Nucleotide differences from Sabin 2 in the VP1 coding region ranged from 12.5 to 15.6 percent, and reached a maximum of 9.6 percent between the VDPV strains under study. Most of the nucleotide substitutions were synonymous but as many as 93 amino acid positions out of 301 in VP1 showed substitutions. We conclude that (1) individuals with prolonged poliovirus infection are not as rare as suggested by the studies on immune deficient patients known to the health care systems and (2) genetic divergence of VDPV strains may remain extensive during years long replication in humans.

## Introduction

The trivalent oral poliovirus vaccine (tOPV) containing the attenuated Sabin strains of the three serotypes of poliovirus (Species *Human enterovirus C*, genus *Enterovirus*, family *Picornaviridae*) has been the central tool in the success of the Global Polio Eradication Initiative (GPEI). The occurrence of new cases of acute poliomyelitis in the world due to wild type poliovirus decreased during the program by more than 99% from 1988 to 2000, but has since then remained between 1000 and 2000 cases annually. Delightfully, year 2012 appeared to end in the lowest number of cases ever (http://www.polioeradication.org; last accessed 19 May, 2013). Wild type 2 poliovirus was eradicated before year 2000, thus all wild type cases during this millennium have been caused by types 1 or 3. The high efficacy of OPV has traditionally overweighed its rare complication, the vaccine associated paralytic poliomyelitis (VAPP) occurring at an incidence of about 1 per half a million primary vaccinations [1], [2], [3]. Another rare complication known for decades is the capacity of the OPV strains to institute prolonged infection in the tissues of persons with a deficiency in the humoral immune systems (reviewed in [1]). During the prolonged infection in an immune deficient host, the vaccine derived poliovirus (VDPV), referred to as iVDPV, is known to drift both genetically and antigenically and not infrequently, to cause a paralytic disease in the host – sometimes several years after the administration of the vaccine [4]. While some VAPP cases were known to occur in unvaccinated contacts of OPV recipients, it was long believed that OPV use cannot initiate poliovirus transmission in human populations. Since the onset of the new millennium, we know it better now. In the absence of the corresponding wild virus serotype and with decreasing OPV coverage, OPV-derived poliovirus strains can circulate in the human population, lose their attenuation and cause paralytic poliomyelitis referred to as outbreaks due to circulating vaccine derived poliovirus (cVDPV), first of its kind discovered in 2000 in the island of Hispaniola [5], [6]. While iVDPV strains usually are much more drifted from the corresponding parental Sabin strain than cVDPV strains, they are genetically of monotypic origin or recombinants of two or three Sabin strains, in contrast to the cVDPV strains which almost always have a part of the non-capsid coding region of the genome derived from one or more unidentified strain(s) of the *Human enterovirus C* (HEV-C) species [3].

Yet another category of VDPV strains has been described, the ambiguous VDPV strains, or aVDPV. They are isolated from healthy persons or patients with non-poliomyelitis disease without an epidemiological connection to paralytic patients, or from the environment. Environmental aVDPVs isolated in several European isolates have shown characteristics of iVDPV strains [3], [7], [8], [9], [10]. Persons shedding these vastly drifted aVDPVs have remained unidentified. Emergence of the different categories of VDPV has caused a change in the plans of necessary polio immunizations after the desired eradication of wild poliovirus transmission. The risks of simple stopping of the use of OPV should be further assessed. In order to design a better policy new data concerning the frequency of generation of VDPV strains as well as about evolution and detailed characteristics of the strains are necessary.

Slovakian Republic, a small Central-European country with decades-long record of elimination of wild poliovirus transmission and no reported wild-type paralytic poliomyelitis since 1960, experienced in 2003–2005 an episode of repeated isolations of environmental aVDPV [11]. Detection of type 2 VDPV in the sewage in the capital Bratislava in April 2003, and subsequently in a distinct township of Skalica, prompted a search for potential virus-shedding immune deficient patient in both regions, without success. In Skalica, the VDPV excretor(s) were localized to an area with a population of about 500 people but an intensive stool survey among them remained negative, and soon thereafter in 2005, all the sewage samples in Skalica also turned negative for VDPV. No VDPVs have been isolated from sewage or clinical specimens in Slovakia since 2005. No clinical cases of poliomyelitis were reported during this aVDPV episode or thereafter.

While the attempts to identify the aVDPV shedding person(s) failed the intensive search provided us with a large number of aVDPV containing sewage samples, and more than 100 VDPV strains isolated over a period of two years. In this paper we report the complete sequence and some phenotypic features of a representative strain, and analyse the overall divergence of 102 unique VP1 sequences.

## Materials and Methods

### Virus strains

Poliovirus strains characterized in this study originated from environmental surveillance for poliovirus in Slovakia, comprising 48 locations throughout the country. The sites were usually sampled bimonthly, thus resulting in more than 280 samples annually. After discovery of the highly drifted strains in Bratislava and Skalica in 2003, the sampling frequency in these locations was intensified as described below. One litre grab samples of raw sewage were collected from one of the main sewers in each location, and processed and concentrated using the two phase separation method essentially as described in [12], [13] at the WHO National Polio Laboratory in Bratislava. The small bottom phase and the so called interphase between the two polymer layers were separately chloroform extracted, and 0.5 ml aliquots of both fractions were inoculated for virus isolation in monolayer cultures of L20B, RD and Hep-2 Cincinnati cells (all authenticated cell lines obtained from the WHO Polio Labnet). Inoculation procedures as well as identification of cytopathic agents were conducted according to the standard techniques recommended by the WHO Polio Labnet. Poliovirus strains were sent for further characterization to the National Institute for Health and Welfare (THL) in Helsinki, where ELISA and restriction fragment length polymorphism (RFLP) were used for intratypic differentiation (ITD) of the strains [14]. Sewage concentrates showing cytopathic effect in any cell line in Bratislava were also sent to Helsinki and re-inoculated in two 50 ml L20B and one RD cell flasks. Hence, aliquots of each sewage specimen were inoculated in 6 to 12 cell culture flasks, and potential isolates from each flasks were processed further individually. All samples received from Bratislava, whether virus isolates or fractions of sewage concentrates, were given a running four-digit code number and any new poliovirus isolates a further processing-step code for unequivocal identification. Poliovirus strains which did not give a Sabin-like result in both ITD tests were subjected to complete VP1 sequencing (see below) to examine the genetic origin of the virus. Only strains intended for complete genomic sequencing or phenotypic characterisation were plaque purified.

### Phenotypic characterisation of selected VDPV isolates

Five VDPV strains from the early phase representing different genetic lineages were selected for phenotypic characterization and tested for antigenic properties by using standard microneutralization assay (WHO Labnet protocol) and sets of human sera drawn from 25 Finnish or 15 Estonian children immunized with at least three doses of trivalent IPV or OPV, respectively. The neurovirulence test was carried out as previously described using PVR-Tg-21 mice [15]. The mice were purchased from the Central Laboratories for Experimental Animals (Kanagawa, Japan). Eight mice (four males and four females) were intracerebrally inoculated for each virus dilution (30 µl/mouse). Ten-fold dilutions of the original virus stock (10^8.0^ CCID_50_/0.1 ml) were prepared (range: 1.48 to 4.48 CCID50/mouse for the MEF-1 strain, 1.48 to 5.48 CCID_50_/mouse for the VDPV strains). The original virus stock 10^7.8^ CCID_50_/0.1 ml) was used for the Sabin 2 strain. Mice were examined daily for 14 days after inoculation, and paralysis or death were recorded. The viral titre that induced paralysis or death in 50% of inoculated mice (PD_50_), was calculated by the method of Kärber and expressed as CCID_50_/mouse. The MEF-1 and Sabin 2 strains were used as virulent and attenuated reference strains.

### Ethical aspects

The anonymised human serum specimens used were from a previous vaccine trial (Finland) or from a seroepidemiological survey (Estonia). Parents of the participants in both studies had given a consent to use the serum sample for medical research. The Estonian specimens were obtained through the courtesy of Dr. Silver Jöks to characterise a previously isolated similar Estonian VDPV strain [7], and made available for similar studies in future. According to the Finnish legislation valid in 2004, the National Public Health Institute (part of the current National Institute for Health and Welfare) had a permission to use old anonymous human specimen collections – without mentioning the country of origin - for public health research. The Law on National Public Health Institute (327/2001, valid from 05-01- 2001 to 12-31-2008) stated as follows:

§1/p2. “The Institute shall monitor, survey, and investigate public health and occurrence of diseases, and participate in explorations and trials aiming at improving public health.”

§1a/p1-3. “The Institute also has the right to collect and process blood and other tissue specimens which are necessary for fulfilling the above purpose. These specimens, as well as similar specimens collected earlier, can be used anonymously for research related to the original purpose of the indicated specimen collection”.

All animal procedures were approved by the Committee for Biosafety and Animal Handling and the Committee for Ethical Regulation of the National Institute of Infectious Diseases, Japan. Animal care, virus inoculation, and observation were performed in accordance with the guidelines of these committees. Mice were sacrificed with carbon dioxide. Every possible effort was made to minimize animal suffering.

### RNA extraction, RT-PCR, and sequencing

Poliovirus strains with aberrant ITD results were subjected to partial genomic sequencing. For that purpose, 100 µl aliquots of culture supernatant were extracted with Rneasy Total RNA Kit (Qiagen), RNA in the VP1 coding part was amplified in RT-PCR, the amplicons were purified by QIAquick Gel Extraction Kit (Qiagen), and sequenced as described before [16]. Complete VP1 encoding region was sequenced from all individual VDPV isolates (excluding some of the very last ones), often several of them originating from a given sewage sample. Partial 3D coding region was sequenced from a proportion of the isolates. The very first isolate PV2-SVK03-E783 was sequenced through the entire genome using a primer walking principle as described before (Table S1) [7]). The GenBank accession numbers of the generated sequences are JX913541 - JX913647 (for VP1 sequences) and JX913648 - JX913690 (for 3D sequences).

### Phylogenetic analysis

Differences and similarities between generated sequences and those downloaded from the GenBank were evaluated with the MEGA software package, versions 4 [17] or 5 [18].

For analysis of genetic relationships of the complete genome of the representative Slovakian VDPV strain PV2-SVK03-E783, PV2 Sabin (AY184220) and all poliovirus and non-polio HEV-C sequences available on 30 March 2012 in GenBank were used. In addition, an unrelated, strongly drifted type 2 VDPV sequence (“PV2-BIR”) was kindly provided by J. Martin, UK [19]. Similarity plotting [20] and Bootscanning [21] analyses of the complete genome alignments were performed with the SimPlot software package (version 2.5 and 3.5.1).

Identification of strains as VDPV was carried out using the complete VP1 sequence and the BLAST program [22] at the NCBI webpage exploiting the GenBank collection of viral sequences. All tested poliovirus type 2 VP1 sequences clustered either very close to that of Sabin 2 (<0.5% difference) or very far from the PV2 Sabin sequence (>12%). The latter were classified as VDPV strains. For phylogenetic analysis, complete VP1 sequences (903 nt) of the VDPV strains were aligned with the Clustal X program [23], together with VP1 sequences of PV2 Sabin, PV2 BIR, and a set of wild PV2 strains (Lansing AY082680, Lederle II AY082678, MEF-1 AY238473, and W2 AY082676). A neighbour-joining (NJ) tree was constructed with the maximum composite likelihood method in the MEGA software package (versions 4 and 5) [17], [18], with internal data-driven correction for transition/transversion bias.

The substituted amino acid positions were placed in the 3-dimensional structure model of poliovirus pentamer based on x-ray crystallographic analysis of type 2 poliovirus strain Lansing (PDB ID: 1IEAH) [24] using Jmol [25].

## Results

### Detection of VDPV strains in sewage samples

**Figure 1:**
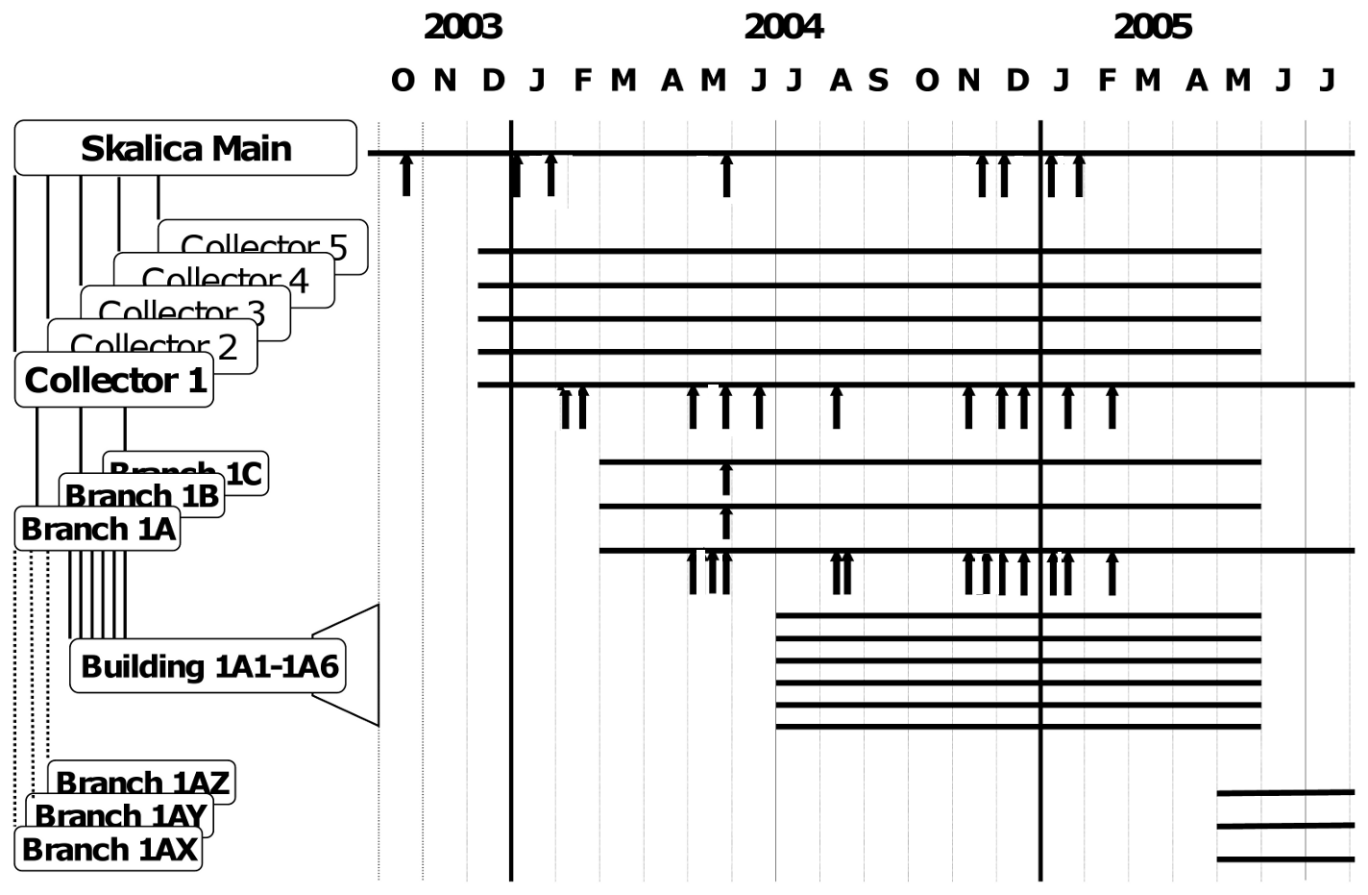
Collection of sewage samples in Skalica 2003–2005. Panel at left shows schematic representation of relevant parts of sewer network in Skalica. The labels and the relation between the sewage collection sites are described in more detail in Results Grab samples from indicated sewers were collected through months covered by horizontal bars (panel at right). Arrows indicate dates of collection of specimens revealing one or more strains of type 2 VDPV in cell culture. See Table 1 for additional details.

The very first VDPV strain, PV2/SVK03/E783, was detected in a sewage sample collected at the Vrakuna sewage plant in Bratislava, April 2003. Intensified sampling in different locations in Bratislava started in June 2003 but only one additional sample, collected in Vrakuna in December, 2003, yielded type 2 poliovirus with VDPV characteristics (#962; in the following the standard prefix, PV2/SVK(year), is replaced by # in the strain identification to simplify the text). An abundant collection of non-polio enterovirus (NPEV) strains was isolated from the samples during the summer and autumn months (Tables S2 and S3). Meanwhile, in October 2003, an ITD-aberrant type 2 poliovirus (#929) genetically related to the first Bratislava VDPV strain had been detected in the inlet sewer of the main sewage treatment plant in Skalica, a small town some 100 km north of Bratislava, close to the border of the Czech Republic [11]. This resulted in intensified sampling not only at the primary sampling site here but - in order to localize the virus excretor(s) - in a form of stepwise walk upstream the network towards smaller and smaller branches with sampling of all incoming sewers at each site of divergence (Fig. 1). In January 2004, the virus was again detected in the inlet of the main municipal sewage treatment plant, and then in February, in one of the five main collector sewers (collector 1). No VDPV strains were isolated in March–April 2004. This coincided with the national annual immunization campaign with OPV, reflected by the isolation of Sabin-like (SL) poliovirus strains from the sewage. In May 2004, the VDPV was again detected in the inlet of the sewage plant, in collector 1, and now also in one of the three sub- branches of collector 1, referred to as sewer branch 1A. On one day, May 28, VDPV strains were exceptionally isolated from the two other sub-branches of collector 1, branches 1B and 1C, as well. On all other days when a VDPV virus was detected in the sewage, the sampling site was one or more locations in the continuum ‘main inlet - collector 1 – branch 1A’. Mutual correlation of VDPV-positive findings at the different levels of the sewerage, and proportions of inoculated cell culture vials yielding the virus are shown in Table 1.

**Table 1:** Coincidence of VDPV detection at different levels of Skalica sewerage.^a^

Date^b^	Sampling site and isolation of VDPV strains in cell culture				
	Number of VDPV yielding vials out of inoculated^c^				
	Skalica main	Collector 1	Branch 1A	Branch 1B	Branch 1C
07-01-2004	**2/12**	0/3	NT	NT	NT
28-01-2004	**3/12**	NT	NT	NT	NT
11-02-2004	0/3	**3/12**	NT	NT	NT
18-02-2004	0/3	**4/12**	NT	NT	NT
12-05-2004	0/3	**2/12**	**1/9**	0/3	0/3
21-05-2004	0/3	0/3	**12/12**	0/3	0/3
28-05-2004	**4/9**	**9/12**	**5/9**	**4/9**	**1/9**
17-06-2004	0/3	**1/9**	0/3	0/3	0/3
05-08-2004	0/3	**1/9**	**1/9**	NT	NT
11-08-2004	0/3	0/3	**1/9**	NT	NT
11-11-2004	0/3	0/3	**2/9**	0/6	0/3
25-11-2004	0/3	**2/9**	0/3	0/3	0/3
30-11-2004	**2/9**	0/3	**2/9**	0/12	0/12
06-12-2004	**3/12**	**4/12**	**6/9**	0/12	0/12
15-12-2004	0/3	**1/9**	**1/9**	0/6	0/3
05-01-2005	**4/12**	**5/12**	0/3	0/3	0/3
13-01-2005	0/3	0/12	**5/12**	0/3	0/3
20-01-2005	**7/12**	0/3	**7/12**	0/3	0/3
17-02-2005	NT	**1/9**	**11/12**	0/3	0/3

a Only dates with at least one VDPV isolated are shown. For details of sewerage structure see Fig. 1;

b Date format: dd-mm-yyyy;

c Bold phase; VDPV-containing samples; some vials yielded more than one strain; NT, not tested.

According to the municipal sewerage plan, branch 1A received all of its sewage from six blocks of flats housing about 500 people altogether. Repeated sampling of the building sewers, and a stool survey with triplicate samples from about a third of the inhabitants, did not yield a single poliovirus. During the summer and early autumn months of 2004 VDPV detections were also less frequent at the main sewage plant, in collector 1 and in branch 1A, but occurred again more regularly from November 2004 to February 2005 (Fig. 1). Relatively large proportion of parallel aliquots of the VDPV-positive sewage samples collected in early 2005 yielded a VDPV strain in cell culture but after February 17, 2005, not a single VDPV strain could be detected in samples collected at any of the sampling sites. In short, one or more type 2 VDPV strains were isolated from 35 sewage samples during a period of 22 months in Slovakia, and altogether 110 strains were separately identified and characterized. No VDPV strain was isolated in the 45 other sampling sites in Slovakia during this episode.

### Complete sequence of the first Slovakia VDPV strain in 2003

The genome of the first Slovakian VDPV isolate, #783, was sequenced entirely as described in the Methods section. The genome contained 7447 nucleotides plus the polyA tail and the typical single open reading frame capable of coding 2207 amino acids. In comparison with sequences available in the GenBank, the closest relative throughout the genome appeared to be the poliovirus type 2 Sabin strain although the distance was remarkable both at the nucleotide and the deduced amino acid levels (Table 2). The 5′UTR of #783 was 3 nucleotides longer than that in the PV2 Sabin strain with a relative 4 cytosine insertion corresponding to sites 97/98 in the PV2 Sabin sequence, and a single nucleotide relative deletion (PV2 Sabin site 137). The four C insertion was not seen in the #977 strain, also sequenced through the 5′UTR. The 3′UTR was 5 nucleotides longer than that in PV2 Sabin with 3 relative insertions, 2 nt at PV2 Sabin site 7408/7409, a single nt at 7429/7430, and a 2 G insertion just before the polyA stretch, at site 7445. Position 481 in the 5′UTR, associated with attenuation of the neurovirulence, had reverted from A in the Sabin 2 to G found in WPV2 strains. The other attenuation associated region coding for amino acid 143 in the VP1 was regularly substituted but was not conserved among the VDPV strains. The *cre* element in the 2C coding region contained 6 substitutions in comparison with the PV2 Sabin sequence. No computer assisted prediction of secondary structure was carried out but as judged from the Fig. 1B of the publication by [26], three of the substitutions could be predicted to stabilize the double stranded stem 1 or 2, one was located in the distal loop and the remaining two could shorten stems 2 and 4 by one base pair each.

**Table 2:** Nucleotide and amino acid identities between PV2/SVK03/783 and PV2Sabin.

Region	Nucleotides			Amino acids		
	Number*	Positions in the alignment	Percent identity	Number#	Positions in polypeptide precursor	Percent identity
**5′UTR**	750	1–751	**88.6**	NR	NR	NR
VP4	207	752–958	88.4	69	1–69	98.6
VP2	813	959–1771	86.5	271	70–340	95.9
VP3	714	1772–2485	83.5	238	341–578	88.2
VP1	903	2486–3388	86.6	301	579–879	94.3
**Entire P1/capsid**	2637	752–3388	**85.9**	879	1–879	**93.5**
2A	447	3389–3835	87.0	149	880–1028	94.6
2B	291	3836–4126	86.6	97	1029–1125	85.6
2C	987	4127–5113	87.5	329	1126–1454	97.9
**Entire P2**	1725	3389–5113	**87.3**	575	880–1454	**95.0**
3A	261	5114–5374	87.0	87	1455–1541	97.7
3B	66	5375–5440	84.9	22	1542–1563	100.0
3C	993	5441–6433	86.7	331	1564–1894	95.2
3D	939	6434–7372	91.0	313	1895–2207	99.0
**Entire P3**	2259	5114–7372	**88.5**	753	1455–2207	**97.2**
**EntireP2+P3**	3984	3389–7372	**87.9**	1328	880–2207	**96.2**
**Entire ORF**	6621	752–7372	**87.1**	2207	1–2207	**95.2**
**3′UTR**	76	7373–7448	**94.0**	NR	NR	NR
**Entire genome**	7448	1–7448	**87.3**	2207	1–2207	**95.2**

* The figures are for SVK783; differences from PV2 Sabin in the 5′UTR and 3′UTR, see the text.

# The length of the ORF was identical in the two strains.

NR, not relevant.

**Figure 2:**
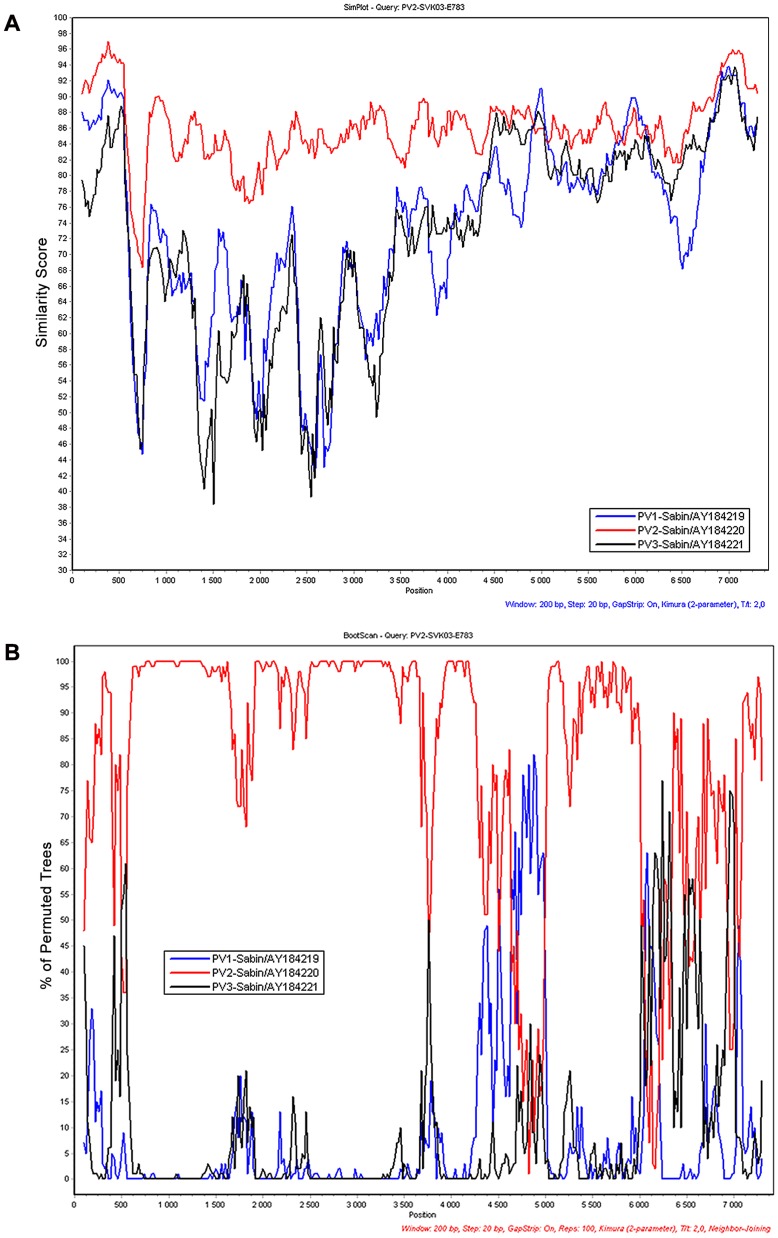
Potential origin of different genome regions of Slovakian VDPV. Simplot (Panel A) [20] and bootscanning (Panel B) [21] analyses of genetic relationship of the index strain of the Slovakian type 2 VDPV episode (strain #783) to the attenuated poliovirus (PV) Sabin strains of all three serotypes. A sliding window of 200 nucleotides with 20 nt step was used. Colour codes: PV1, blue; PV2, red; PV3, black.

A Simplot –analysis of genetic relationships of the #783 strain to other poliovirus and non-polio HEV-C strains (not shown) suggested PV2 Sabin as the closest relative throughout the genome, except two short stretches at positions about 5000 and 6000, respectively, which appeared to be closer to PV1 Sabin than to PV2 Sabin (Fig. 2). A closer look at variable sites in the sequence revealed more PV1 Sabin -like than PV2 Sabin -like nucleotide sites in the #783 sequence in both regions, but no crossing points could be identified as several intervening sites where unique for #783 differing from both Sabin 2 and Sabin 1, a few of these even so that the latter two were identical (data not shown).

**Figure 3:**
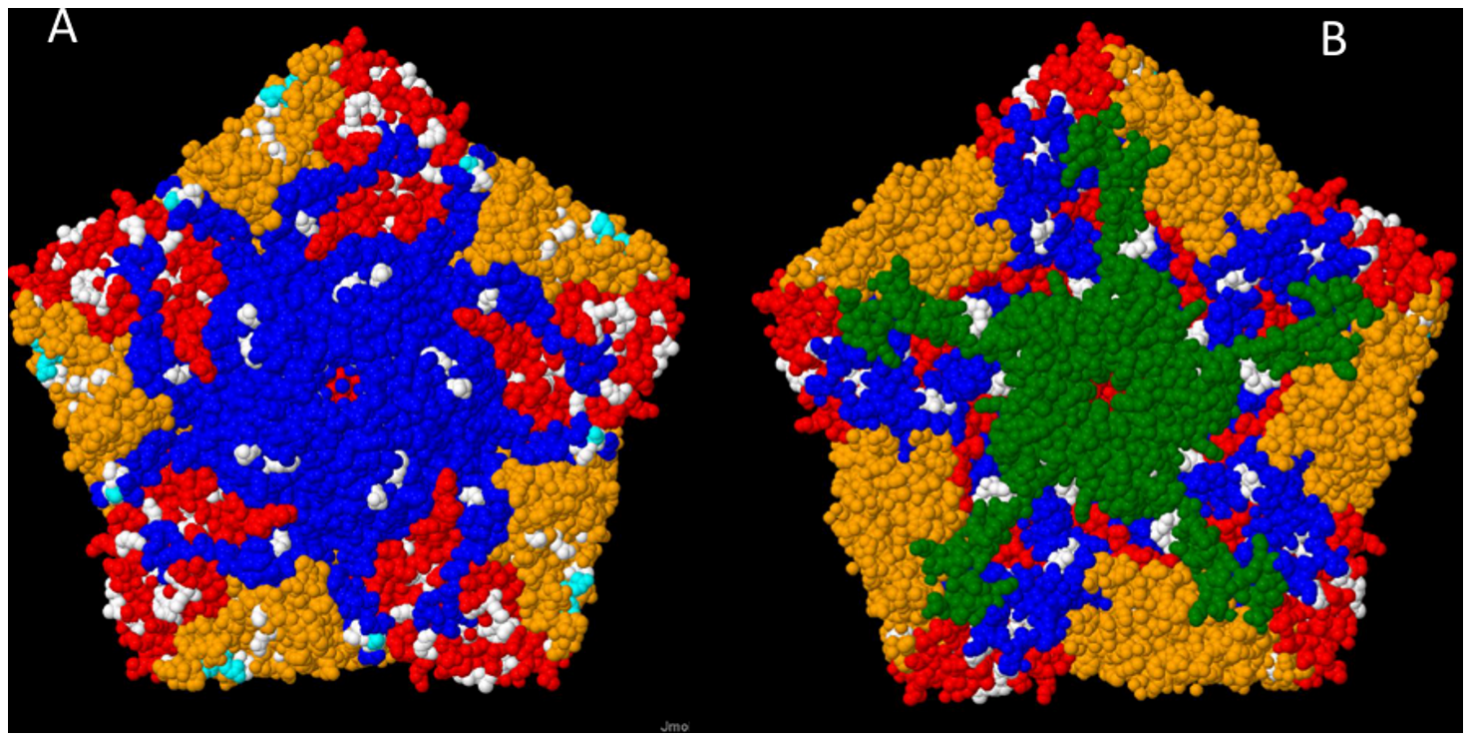
Location of substituted amino acid sites in 3-dimensional structure model of poliovirus capsid protein pentamer. Visualisation is based on x-ray crystallographic analysis of type 2 poliovirus strain Lansing (PDB ID: 1IEAH) [24]. Panel A, view from outside of virion; Panel B, view from inside the capsid wall. Locations of amino acids substituted in strain #783 in comparison to Sabin 2 are indicated. Colour codes: VP1, blue; VP2, orange; VP3, red;VP4, green. Substitutions at known antigenic sites, cyan. Substitutions elsewhere, white. Note that the BC-loop of VP1 is not visible in this model.

Alignment of the #783 amino acid sequence with those of prototype poliovirus strains suggested full conservation of the cleavage sites of the viral polypeptide (data not shown), numerous substitutions in the known antigenic sites of Sabin 2 (Table 3; Fig. 3), but also elsewhere throughout all proteins (Table 2). The overall amino acid identity with Sabin 2 was 95.2% with greatest similarity in the P3 region. The most divergent proteins were 2B and VP3. In the 2B a large part of the divergence came from an eight amino acid motive, EMVSVIIN, which showed identities of only 1–2 amino acids with any of the three Sabin strains and, according to a BLAST search (26 March, 2012), did not resemble sequences of any other enteroviruses either.

**Table 3:** Amino acid substitutions in Slovakian VDPV strain #783 at different antigenic sites.

	Antigenic sites, amino acid sites and sequence							
Virus strain	Site 1	Site 2		Site 2b	Site 3	Site 3a		Site 3b
	VP1 91-105	VP1 221-226	VP2 162-172	VP2 72	VP1 287-291	VP3 58-62	VP3 70-75	VP3 76-80
PV2Sabin	EVDNDAPTKRASRLF	ASTEGD	LDTNATNPARN	R	KDGLT	TSQRR	VELSDT	AHSDT
PV2-MEF-1	------------K--	------	-----------	-	----A	----K	---N-A	-----
#783	--------Q--AK--	SPS---	--------T--	N	-----	-N---	--MR-P	-PT--

Antigenic sites are according to [10]. Differences from PV2 Sabin are indicated by one letter code of amino acid; -, identical with PV2Sabin.

### Neurovirulence and antigenic properties of selected VDPV strains

Two Slovakian VDPV strains (#783 and #929) were plaque-purified and tested for neurovirulence using standard techniques in the PVR- expressing recombinant mouse model [15]. Both strains showed increased neurovirulence as compared to the type 2 Sabin strain, although neither of them reached the level of a reference WPV2 strain (Table 4).

**Table 4:** Neurovirulence of selected Slovakian type 2 VDPV strains in PVR-transgenic mouse model.

		Inoculated virus titre (log[CCID_50_/0.1 ml]) (30 µl/mouse)							
Virus	Initial virus titer	Mortality							PD_50_
	(log[CCID_50_/0.1 ml])	7.28	7.03	5.48	4.48	3.48	2.48	1.48	
PV2 Sabin	7.8	0/8	NT	NT	NT	NT	NT	NT	>7.28
MEF-1 (PV2-wild)	8.0	NT	NT	NT	6/6	6/8	3/8	0/8	2.79
#SVK783	8.0	NT	NT	7/8	1/8	3/8	0/8	0/8	≤4.64
#SVK929	8.0	NT	NT	6/8	3/8	0/8	0/8	0/8	≤4.81

PD_50_, paralytic dose-50.

NT, not tested.

The above two strains and single plaque lineages of three other strains (#962, #975, and #977) were tested for antigenic properties using sets of human immune sera in the standard microneutralization assay. Twenty five Finnish sera from IPV immunized children and 15 Estonian sera from OPV immunized children all had neutralizing antibodies to PV2 Sabin at varying levels. All sera were also capable of neutralizing all the five VDPV strains but the titres against PV2 Sabin were usually 2-fold or sometimes 4-fold higher (data not shown).

### Phylogenetic analysis of the 110 VP1 coding sequences from Slovakian VDPV strains

One hundred and ten VDPV strains were sequenced through the VP1 coding region, and the sequences were subjected to genetic analysis in MEGA5. No deletions or insertions were found within this 903 nt region. As mentioned in the preliminary publication [11], the VP1 sequences of the SVK VDPV strains differed from that of PV2 Sabin at 113–141 nt sites corresponding to p-differences from 12.5 to 15.6 per cent. The overall diversity of the VDPV sequences was vast, with a maximum p-difference of 9.6 per cent representing mutual substitutions at 87 nucleotide sites (strains #1398-Z1 and #1508). Out of the 110 sequenced strains, four small sets of identical sequences were seen. For two of the sets the strains were derived from two different sewage samples each but in both cases the samples had been collected on the same day, respectively. In further analysis, only one representative of each repetitive set of sequences was used reducing the number of Slovakian VDPV strains to 102.

**Figure 4:**
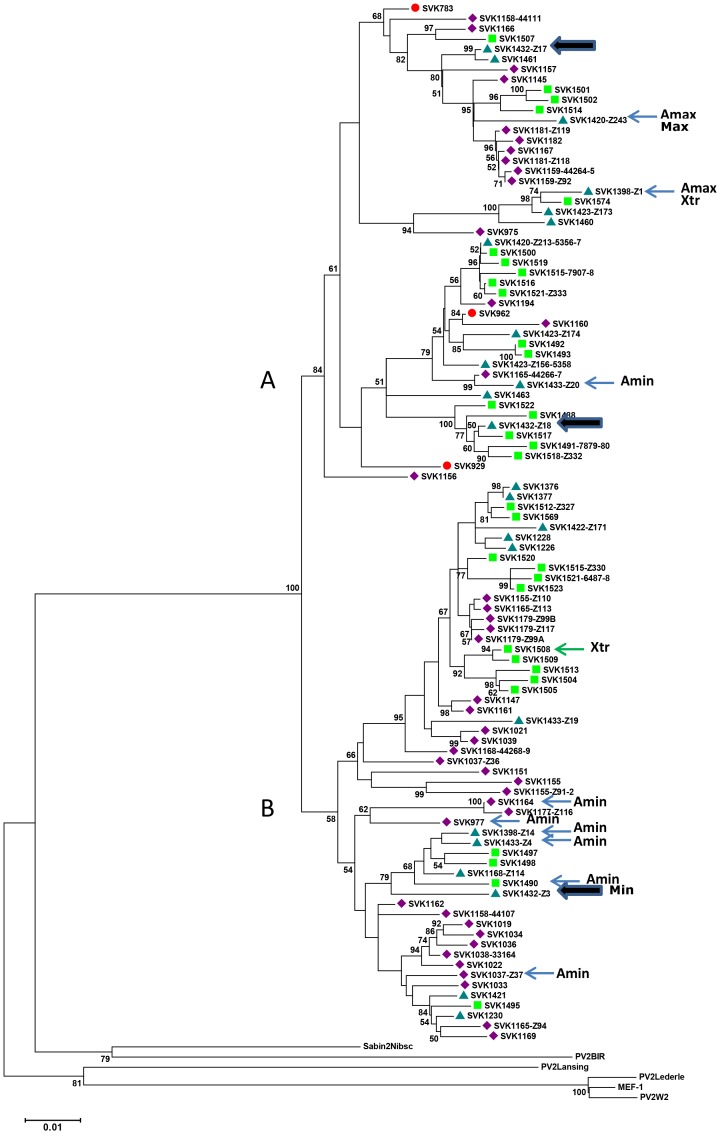
Phylogenetic analysis of VP1 sequences of 102 Slovakian type 2 VDPV strains. A set of selected type 2 poliovirus reference strains is used as outgroup. Evolutionary distances were computed using the Maximum Composite Likelihood method and Neighbour-Joining tree, consensus from 1000 bootstrap replicates, is shown. Bootstrap values smaller than 50% are omitted. The coloured symbols indicate time of sample collection: red circle, 2003; purple diamond, January–June 2004; blue triangle, July–December 2004; green square, 2005. Large arrows indicate an example of divergent strains isolated from a single sewage specimen. Strains with maximum and minimum nucleotide p-distances from poliovirus 2 Sabin are indicated by Max and Min, respectively. Two strains showing the maximum p-distance between two VDPV strains are labelled by Xtr. Strains showing the largest and smallest difference in amino acids from Sabin 2 are shown by Amax and Amin labels, respectively.

In phylogenetic analysis conducted using the maximum composite likelihood algorithm and including reference sequences of other type 2 poliovirus strains, all 102 unique SVK VDPV sequences were found to be monophyletic descending from PV2 Sabin. An NJ tree constructed allowing gamma-distributed rates for sites, and heterogeneous rates among lineages, is shown in Fig. 4. The sequences segregated in two major clusters (A and B) with high or moderate bootstrapping support. Further division of the major clusters to 2–3 sub-clusters was moderately supported. Analysis made by using PV2 Sabin alone as a root did not change the overall branching order but made some of the sub-cluster divisions less well supported (not shown).

Representatives of both major clusters were detected throughout the follow up period. Frequently, a given sewage specimen contained strains belonging to different sub-clusters (strains isolated from sample #1432 indicated as an example in Fig. 4 by the large arrows). The three strains detected in samples collected in the early phase of the episode (year 2003, indicated by filled red circles in Fig. 4) had positions relatively close to the PV2 Sabin root in the tree. However, two other strains, #1162 and #1156, isolated several months later, appeared to be as close to Sabin 2 as the very first strain #783 (Fig. 4). None of the five strains mentioned above showed the smallest relative nucleotide p-difference from Sabin 2. The minimum value 12.5 per cent was recorded for strain #1432-Z3 isolated from a specimen collected during the second half of 2004. In the tree, this strain was not located very close to PV2 Sabin. Strains isolated from samples collected during the late stage of the episode, year 2005 (green squares in Fig. 4), often located at the right hand far away tips of the lineages but were accompanied by some strains originating from the second or even the first half of the year 2004 samples. The strain #1420-Z243 had the largest p-difference (15.6 per cent) in nucleotide sequence and was also one of the two strains most divergent from PV2 Sabin in amino acid sequence (25 out of 301 amino acid sites). The seven strains showing the smallest amino acid difference from PV2 Sabin (14 amino acids) did not include any of the above mentioned strains which were phylogenetically relatively close to PV2 Sabin (Fig. 4). Most of the nucleotide diversity in VP1 was silent and altogether 204 deduced amino acid sites out of the 301 were fully conserved when compared with the VP1 sequence of PV2 Sabin.

## Discussion

In this paper we have described details of a previously reported episode of extended occurrence of neurovirulent type 2 VDPV strains in environmental specimens collected in Slovakia. The episode was not associated with reported cases of paralytic poliomyelitis, and detection of VDPV strains in the sewage ended without any intervention. However, we do not know whether the prolonged poliovirus excretion really ended, or whether the person(s) excreting the virus moved to another location, or died. Sequences coding for capsid protein VP1 were determined for 110 isolates covering a period of 22 months. All were monophyletic originating from PV2 Sabin but had subsequently evolved showing vast sequence difference to PV2 Sabin and also wide divergence between each other.

### Significance of aVDPV strains detected in environmental but not in clinical specimens

Isolation of strongly drifted VDPV strains from sewage specimens is not a rare finding although so far, most of the characterized environmental aVDPV strains have been detected in a limited number of countries [7], [8], [10]. We believe that this limitation is more likely due to the systematic use of high coverage environmental surveillance for poliovirus circulation in these countries rather than reflecting true differences between human populations in the prevalence of individuals with prolonged poliovirus infection. In spite of intensive search, the excretor(s) of these aberrant viruses have remained undisclosed, even in cases of several repeated isolations of related VDPV strains. So was also the case with the type 2 VDPV strains characterized in this paper. Like in the previously described extended episodes of aVDPV occurrence in sewage specimens, no cases of poliomyelitis were reported in source population or elsewhere in Slovakia over several years preceding, during or, until now, after isolation of these aVDPV strains. Prolonged occurrence of neurovirulent and antigenically drifted poliovirus strains in the environment is, however, a signal of potential risk of transmission of pathogenic poliovirus in the source population. Most likely, high vaccination coverage of the Slovakian population prevented wider spreading of the virus during this episode. It is noteworthy that the virus content in the VDPV positive sewage samples appeared to be relatively high in January–February 2005, and then the occurrence of VDPV strains suddenly stopped. This suggests that the person(s) shedding the virus probably moved to another location rather than stopped shedding the virus.

From the point of view of the global eradication of poliomyelitis, accumulation of episodes with presence of iVDPV resembling aVDPV strains for extended periods in the environment indicates that individuals presenting with prolonged poliovirus excretion may not be as rare as judged from known cases of immune deficient patients [27]. Development of antiviral drugs to stop prolonged poliovirus excretion has been encouraged and some progress towards this direction has been reported [28]. However, if the excretors are not known, even very effective drugs are likely to be useless. Systematic environmental surveillance for poliovirus should be widely extended and intensified in order to obtain a realistic view on the prevalence of prolonged poliovirus excretion.

### One VDPV excretor or also abortive spreading to close contacts?

All the VDPV strains characterized in this study were monophyletic and all the lineages most likely originally derived from a single vaccinee. However, it is open to debate, whether all the strains detected during the episode actually were shed by a single person, or by a few persons, as we could not identify the source of the virus strains. Therefore, potential spreading of the virus to close contacts of the main excretor remains a speculative possibility, although all the observations also fit to the alternative that only a single person was excreting the virus. First, while related VDPV strains were isolated from two separate towns, two strains from Bratislava and the rest from Skalica, the dates of VDPV-positive sewage sample collections did not overlap. A person living in Skalica might well have visited the capital twice during the episode. In Skalica, all but two of the VDPV-positive sewage samples had been collected in sites that, within the sewage network, formed a linear continuum from the suspected 500-people neighbourhood to the main sewage plant of the town. The two aberrant sewage specimens had been collected on a single day when also the more regularly positive parallel sewer branch was found to be relatively strongly positive for the VDPV. A putative laboratory contamination is an unlikely explanation as the “aberrant” sewage specimens also contained unique VDPV variants not found elsewhere. Complexity of a sewerage is known to be able to delay by several days part of the sewage flow and thus also to increase the “washing-out” time of a poliovirus bolus introduced into the sewage [29]. Hence, finding of VDPV strains on a given day in three independent sewers does not necessarily mean that the putative single shedder should have been using toilets of the three adjacent but independent sewage catchment areas on the same day. It is worth mentioning, however, that the sampling sites in question were rather close to the corresponding housing areas and thus the delay of sewage flow most likely was significantly less than that in the cited Helsinki study [29]. Therefore, a possibility also remains that VDPV strains found in these aberrant sampling sites might represent abortive spreading of the VDPV strains to contacts of the main excretor living in other areas in Skalica.

### Characteristics of the Slovakian type 2 VDPV strains

Complete genomic sequence of the first detected strain of the episode revealed vast divergence from the sequence of the Sabin 2 vaccine strain throughout the genome. Comparison of the VDPV nucleotide sequence with those of other HEV-C strains in GenBank did not reveal any signs of recombination with non-polio HEV-C strains in the evolutionary history of the Slovakian type 2 VDPV strain, a finding typical of previously described aVDPV strains with iVDPV-like degree of genetic divergence [7], [8]. However, Simplot-analysis suggested, and nucleotide by nucleotide examination confirmed, that at two relatively short stretches in the non-structural protein coding region of the genome the VDPV sequence was closer to Sabin 1 than to Sabin 2 sequence. Frequent unique substitutions both within the stretches and in the flanking regions did not allow justified definition of possible junction points of the putative intertypic poliovirus recombination and thus, this possibility remained unconfirmed. Nucleotide substitutions known to be associated with loss of attenuation and reversion to neurovirulence of the PV2 Sabin strain were found in the SVK VDPV strains. The two strains tested for neurovirulence in the transgenic mouse model also revealed a capacity definitely greater than that of Sabin 2 to induce paralytic disease, similar to previous observations on vastly drifted aVDPV strains [9], [10].

While most of the nucleotide differences between Slovakian VDPV and PV2 Sabin were silent, deduced amino acid substitutions were also frequent and ranged both structural and non-structural proteins. Yet, five representative Slovakian VDPV strains tested were all readily neutralised by sera containing antibodies induced either by OPV or IPV in children. The antibody titres, however, were throughout lower than those against the PV2 Sabin strain suggesting that these neurovirulent strains might have a spreading potential in sub-optimally immunized human populations. High vaccination coverage of the Slovakian population most likely prevented the spreading of the virus in this incident in spite of prolonged excretion.

### Vast genetic divergence but only tips of icebergs analysed

Phylogenetic analysis of the VP1 sequences of 110 environmental isolates over a sampling time of almost two years revealed a vast divergence of the sequences and segregation into several coexisting lineages. In poliovirus evolution, genetic lineages are generated already during the acute infection of a single host individual [30] and previous reports on iVDPV excretion have shown that in prolonged poliovirus infection, several coexisting lineages may persist for variable periods [31], [32]. The frequency of collection of grab samples of the sewage varied on different time points and at different locations. Furthermore, assuming only a single VDPV excretor, the poliovirus isolation attempts may have been carried out below or close to the detection limit, in spite of a fair overall sensitivity of the Slovakian laboratory reflected by repeated isolations of non-polio enteroviruses and adenoviruses. In acute poliovirus infection a person is known to excrete usually not more than 10^7^ CCID_50_ per day [33]. In prolonged infection the daily amount may be lower than the peak level of acute infection. Assuming a daily sewage flow in the sewerage of 100 litres per person per day or a mean daily flow rate of 1.7 million litres for the 17 000 Skalica population. A further assumption of about 50-fold concentration of the sewage specimen in the laboratory would result to about 0.3 CCID_50_ VDPV/ml of cell culture inoculum. This is fitting well to the observation that only some of the main inlet grab samples were positive for VDPV. A similar calculation for branch 1A with the assumed source population of less than 1000 people would suggest a maximal VDPV concentration of about 10 CCID_50_/ml of the inoculum. This amount should be regularly detected in cell culture but grab sampling of sewage this close to the excretor's home is affected by the time of toilet usage and thus, a single grab sample is likely to be suboptimal in sensitivity. In brief, in spite of the large number of VDPV strains with vast genetic divergence we have analysed only the tips of icebergs of all possible Slovakian VDPV variants. It is noteworthy that usually the VDPV strains were isolated only from a part of the several cell culture vials inoculated with aliquots of the concentrate of a given sewage specimen. While this observation suggests that individual isolates most likely were rather homogeneous, it also means that we could characterise only the majority populations of the variants at most of the time points. Anyhow, no sign of reduction of genetic divergence over time was seen, an observation at variance with that reported in one previous publication [31].

### Final remarks

A 22 months period of repeated isolations of type 2 VDPV from Slovakian sewage ended without intervention and caused no paralytic cases of poliomyelitis. However, we do not know if the shedding really stopped or whether the person(s) responsible for the occurrence of the VDPV strains in sewage, moved to another location. While both the genetic and the phenotypic properties of the VDPV strains resembled those of iVDPV, the person(s) responsible for this continuous shedding of VDPV into environment remained unidentified. This indicates that the prevalence of individuals with prolonged poliovirus infection is greater than judged from the studies of immune deficient patients known to the health care systems. This view is important to consider when designing post-eradication immunization policies. The characterized VDPV strains presented with persisting vast genetic divergence and, at least in the VP1, also showed a wide range of amino acid substitution in several domains of the protein. Further divergence within the lineages appeared to be generated during the 22 months observation period and is a topic of a separate analysis.

## Supporting Information

Table S1 **Primers used for the complete genome sequencing.**
(DOC)

Table S2 **Monthly detection of vaccine derived and Sabin-like polioviruses and non-polioviruses in Skalica 2003–2005.**
(DOC)

Table S3 **Number of sewage samples positive for different cytopathic viruses.**
(DOC)
