# Supplementary material for: Characteristics of an Environmentally Monitored Prolonged Type 2 Vaccine Derived Poliovirus Shedding Episode that Stopped without Intervention
Source: PLoS One. 2013 Jul 31;8(7):e66849. doi: 10.1371/journal.pone.0066849 (PMC3729856; doi:10.1371/journal.pone.0066849)
Supplement: Table S1 — Primers used for the complete genome sequencing. (DOC) [file pone.0066849.s001.doc]

**Table S1.** Primers used for the complete genome sequencing

| Primer | Position in PV2 Sabin | Orientation | Sequence (5' - 3') |
| --- | --- | --- | --- |
| SVK-5NCRFIII | 1-15 | sense | TTA AAA CAG CTC TGG |
| SVK-5NCRF | 1-18 | sense | TTA AAA CAG CTC TGG GGT |
| SVK-5NCRFII | 356-374 | sense | AGG CTG CGT TGG CGG CCT A |
| SVK-VP4R | 787-769 | antisense | CGT GGG CTC CCA CTT TCT G |
| SVK-VP2FII | 1066-1084 | sense | TGG CCT GAA TAC ATT AGA G |
| SVK-VP2R | 1308-1291 | antisense | ATG AAA CTT TGA AGC ATT |
| SVK-VP2F | 1719-1736 | sense | GTG TTG CGA ATT CAA TGG |
| SVK-VP3R | 2423-2404 | antisense | TCA TTG CAA GCT GAC ACA AA |
| SVK-VP1FII | 2920-2939 | sense | CGG ACA TGC ATT GAA CCA GG |
| SVK-VP1F | 3214-3230 | sense | GTG AAT GAT CAT AAT CC |
| SVK-2CR | 4445-4429 | antisense | ATG GTA TGC TCC AGC TT |
| SVK-2CRII | 4502-4483 | antisense | ACT AAC AAG CAT ACT GGC TC |
| SVK-2CFII | 4942-4955 | sense | CAA CCA GCA AAC TT |
| SVK 2CF | 5000-5017 | sense | GGA CAA GTC TTC CAG GGT |
| SVK-3DRII | 6095-6076 | antisense | ACC CCT TCA AAC ACA TAG TG |
