# Supplementary material for: Characteristics of an Environmentally Monitored Prolonged Type 2 Vaccine Derived Poliovirus Shedding Episode that Stopped without Intervention
Source: PLoS One. 2013 Jul 31;8(7):e66849. doi: 10.1371/journal.pone.0066849 (PMC3729856; doi:10.1371/journal.pone.0066849)
Supplement: Table S2 — Monthly detection of vaccine derived and Sabin-like polioviruses and non-polioviruses in Skalica 2003–2005. (DOC) [file pone.0066849.s002.doc]

**Table S2.** Monthly detection of vaccine derived and Sabin-like polioviruses and non-polioviruses in Skalica 2003 - 2005

|  |  | Month (two letter code) followed by year (200x) and number of samples positive for indicated virus group or negative for any cytopathic virus | | | | | | | | | | | | | | | | | | | | | | | | | | | |
| --- | --- | --- | --- | --- | --- | --- | --- | --- | --- | --- | --- | --- | --- | --- | --- | --- | --- | --- | --- | --- | --- | --- | --- | --- | --- | --- | --- | --- | --- |
| Site | Virus | Ap3 | My3 | Jn3 | Jl3 | Au3 | Se3 | Oc3 | No3 | De3 | Ja4 | Fe4 | Mr4 | Ap4* | My4 | Jn4 | Jl4 | Au4 | Se4 | Oc4 | No4 | De4 | Ja5 | Fe5 | Mr5 | Ap5 | My5 | Jn6 | Jl5 |
| **Bra** | **VD** | **1** |  |  |  |  |  |  |  | **1** |  |  |  |  |  |  |  |  |  |  |  |  |  |  |  |  |  |  |  |
|  | SL | 1 | 1 |  |  |  |  |  |  |  |  |  |  |  |  |  |  |  |  |  |  |  |  |  |  | 1 |  |  |  |
|  | NP |  |  | 1 | 2 | 6 | 1 | 1 |  | 1 |  |  |  |  |  |  |  |  | 1 |  | 1 |  |  |  |  | 1 |  |  |  |
|  | Negat |  |  | 1 | 3 |  | 1 | 1 | 2 | 1 | 2 |  | 5 | 1 | 1 | 1 | 1 |  |  |  |  |  | 1 |  |  | 1 |  | 1 | 1 |
| **Ska M** | **VD** |  |  |  |  |  |  | **1** |  |  | **2** |  |  |  |  |  |  |  |  |  | **1** | **1** | **2** |  |  |  |  |  |  |
|  | SL |  |  |  |  |  |  |  |  |  |  |  | 1 |  |  |  |  |  |  |  |  |  |  |  |  |  |  |  |  |
|  | NP |  | 1 |  |  | 1 |  | 2 |  |  | 1 |  |  |  |  |  | 1 | 1 |  | 1 | 1 |  |  |  |  | 1 |  |  | 1 |
|  | Negat |  |  |  |  |  |  |  |  | 2 | 2 | 4 | 3 | 4 | 3 | 4 | 4 | 3 | 2 | 2 | 3 | 1 |  |  |  |  |  | 2 | 3 |
| **Coll 1** | **VD** |  |  |  |  |  |  |  |  |  |  | **2** |  |  | **2** | **1** |  | **1** |  |  | **1** | **2** | **1** | **1** |  |  |  |  |  |
|  | SL |  |  |  |  |  |  |  |  |  |  |  |  |  |  |  |  |  |  |  |  |  |  |  |  |  |  |  |  |
|  | NP |  |  |  |  |  |  |  |  |  | 1 |  |  |  |  |  | 1 |  | 1 |  |  |  |  | 1 |  |  |  |  | 1 |
|  | Negat |  |  |  |  |  |  |  |  | 1 |  | 2 | 4 | 4 | 2 | 3 | 4 | 3 | 1 | 2 | 3 |  | 1 | 2 | 1 | 2 | 3 | 4 | 3 |
| **Coll 2** | VD |  |  |  |  |  |  |  |  |  |  |  |  |  |  |  |  |  |  |  |  |  |  |  |  |  |  |  |  |
|  | SL |  |  |  |  |  |  |  |  |  |  |  | 1 |  |  |  |  |  |  |  |  |  |  |  |  |  |  |  |  |
|  | NP |  |  |  |  |  |  |  |  |  | 1 |  |  |  |  |  |  |  |  |  |  |  |  |  |  |  |  |  |  |
|  | Negat |  |  |  |  |  |  |  |  | 1 |  | 4 | 3 | 4 | 4 | 4 | 3 |  |  | 2 | 4 | 1 | 2 | 2 | 1 | 2 | 2 |  |  |
| **Coll 3** | VD |  |  |  |  |  |  |  |  |  |  |  |  |  |  |  |  |  |  |  |  |  |  |  |  |  |  |  |  |
|  | SL |  |  |  |  |  |  |  |  |  |  |  |  | 1 |  |  |  |  |  |  |  |  |  |  |  |  |  |  |  |
|  | NP |  |  |  |  |  |  |  |  |  |  |  |  |  | 1 |  | 1 |  |  |  | 2 |  |  | 1 |  |  |  |  |  |
|  | Negat |  |  |  |  |  |  |  |  | 1 | 1 | 4 | 4 | 3 | 3 | 4 | 2 |  |  | 2 | 2 | 2 | 2 | 2 | 1 | 2 | 2 |  |  |
| **Coll 4** | VD |  |  |  |  |  |  |  |  |  |  |  |  |  |  |  |  |  |  |  |  |  |  |  |  |  |  |  |  |
|  | SL |  |  |  |  |  |  |  |  |  |  |  |  |  |  |  |  |  |  |  |  |  |  |  |  |  |  |  |  |
|  | NP |  |  |  |  |  |  |  |  |  |  |  |  |  | 1 |  |  |  |  |  | 1 |  |  |  |  |  |  |  |  |
|  | Negat |  |  |  |  |  |  |  |  | 1 | 1 | 4 | 4 | 4 | 3 | 4 | 3 |  |  | 2 | 3 | 1 | 2 | 2 |  | 2 | 2 |  |  |
| **Coll 5** | VD |  |  |  |  |  |  |  |  |  |  |  |  |  |  |  |  |  |  |  |  |  |  |  |  |  |  |  |  |
|  | SL |  |  |  |  |  |  |  |  |  |  |  | 1 | 6 | 3 |  |  |  |  |  |  |  |  |  |  |  |  |  |  |
|  | NP |  |  |  |  |  |  |  |  |  | 1 |  |  | 1 |  |  |  |  |  |  | 1,4 | 1 |  |  |  |  |  |  |  |
|  | Negat |  |  |  |  |  |  |  |  | 1 |  | 4 | 3 | 3 | 2 | 3 |  |  |  | 2 |  | 1 | 2 | 2 | 1 | 2 | 2 |  |  |
| **Branch** | **VD** |  |  |  |  |  |  |  |  |  |  |  |  |  | **3** |  |  | **2** |  |  | **2** | **2** | **2** | **1** |  |  |  |  |  |
| **1A** | SL |  |  |  |  |  |  |  |  |  |  |  |  |  | 1 | 1 |  |  |  |  |  |  |  |  |  |  |  |  |  |
|  | NP |  |  |  |  |  |  |  |  |  |  |  |  |  |  |  | 1 |  |  |  | 1 |  |  |  |  |  |  |  |  |
|  | Negat |  |  |  |  |  |  |  |  |  |  |  | 1 | 4 | 1 | 3 | 4 | 3 | 2 | 3 | 1 |  | 1 | 1 | 1 | 2 | 3 | 4 | 3 |
| Build | VD |  |  |  |  |  |  |  |  |  |  |  |  |  |  |  |  |  |  |  |  |  |  |  |  |  |  |  |  |
| 1A1 | SL |  |  |  |  |  |  |  |  |  |  |  |  |  |  |  |  |  |  |  |  |  |  |  |  |  |  |  |  |
|  | NP |  |  |  |  |  |  |  |  |  |  |  |  |  |  |  |  |  | 1, 2 | 2 |  |  |  |  |  |  |  |  |  |
|  | Negat |  |  |  |  |  |  |  |  |  |  |  |  |  |  |  | 2 | 4 |  | 2 | 3 | 2 | 2 | 1 |  | 1 | 1 |  |  |
| Build | VD |  |  |  |  |  |  |  |  |  |  |  |  |  |  |  |  |  |  |  |  |  |  |  |  |  |  |  |  |
| 1A2 | SL |  |  |  |  |  |  |  |  |  |  |  |  |  |  |  |  |  |  |  |  |  |  |  |  |  |  |  |  |
|  | NP |  |  |  |  |  |  |  |  |  |  |  |  |  |  |  | 1 |  | 4 | 1 | 1 |  |  |  |  |  |  |  |  |
|  | Negat |  |  |  |  |  |  |  |  |  |  |  |  |  |  |  | 1 | 4 | 1 | 3 | 2 | 2 | 2 | 1 | 1 | 2 | 1 |  |  |
| Build | VD |  |  |  |  |  |  |  |  |  |  |  |  |  |  |  |  |  |  |  |  |  |  |  |  |  |  |  |  |
| 1A3 | SL |  |  |  |  |  |  |  |  |  |  |  |  |  |  |  |  |  |  |  |  |  |  |  |  |  |  |  |  |
|  | NP |  |  |  |  |  |  |  |  |  |  |  |  |  |  |  |  |  | 2 | 2 |  |  |  |  |  |  |  |  |  |
|  | Negat |  |  |  |  |  |  |  |  |  |  |  |  |  |  |  | 2 | 4 | 3 | 2 | 3 | 2 | 2 | 1 | 1 | 2 | 1 |  |  |
| Build | VD |  |  |  |  |  |  |  |  |  |  |  |  |  |  |  |  |  |  |  |  |  |  |  |  |  |  |  |  |
| 1A4 | SL |  |  |  |  |  |  |  |  |  |  |  |  |  |  |  |  |  |  |  |  |  |  |  |  |  |  |  |  |
|  | NP |  |  |  |  |  |  |  |  |  |  |  |  |  |  |  |  |  | 1 |  |  |  |  |  |  |  |  |  |  |
|  | Negat |  |  |  |  |  |  |  |  |  |  |  |  |  |  |  | 2 | 4 | 4 | 4 | 3 | 2 | 2 | 1 | 1 | 2 | 1 |  |  |
| Build | VD |  |  |  |  |  |  |  |  |  |  |  |  |  |  |  |  |  |  |  |  |  |  |  |  |  |  |  |  |
| 1A4.1 | SL |  |  |  |  |  |  |  |  |  |  |  |  |  |  |  |  |  |  |  |  |  |  |  |  |  |  |  |  |
|  | NP |  |  |  |  |  |  |  |  |  |  |  |  |  |  |  |  |  |  |  | 1 | 1 |  |  |  |  |  |  |  |
|  | Negat |  |  |  |  |  |  |  |  |  |  |  |  |  |  |  |  |  | 4 | 2 | 2 | 1 | 1 |  |  |  |  |  |  |
| Build | VD |  |  |  |  |  |  |  |  |  |  |  |  |  |  |  |  |  |  |  |  |  |  |  |  |  |  |  |  |
| 1A5 | SL |  |  |  |  |  |  |  |  |  |  |  |  |  |  |  |  |  |  |  |  |  |  |  |  |  |  |  |  |
|  | NP |  |  |  |  |  |  |  |  |  |  |  |  |  |  |  |  |  | 1 |  |  |  |  |  |  |  |  |  |  |
|  | Negat |  |  |  |  |  |  |  |  |  |  |  |  |  |  |  | 2 | 4 | 4 | 4 | 3 | 2 | 2 | 1 | 1 | 2 | 1 |  |  |
| Build | VD |  |  |  |  |  |  |  |  |  |  |  |  |  |  |  |  |  |  |  |  |  |  |  |  |  |  |  |  |
| 1A5.1 | SL |  |  |  |  |  |  |  |  |  |  |  |  |  |  |  |  |  |  |  |  |  |  |  |  |  |  |  |  |
|  | NP |  |  |  |  |  |  |  |  |  |  |  |  |  |  |  |  |  |  |  |  |  |  |  |  |  |  |  |  |
|  | Negat |  |  |  |  |  |  |  |  |  |  |  |  |  |  |  |  |  | 4 | 2 | 3 | 2 | 1 |  |  |  |  |  |  |
| Build | VD |  |  |  |  |  |  |  |  |  |  |  |  |  |  |  |  |  |  |  |  |  |  |  |  |  |  |  |  |
| 1A6 | SL |  |  |  |  |  |  |  |  |  |  |  |  |  |  |  |  |  |  |  |  |  |  |  |  |  |  |  |  |
|  | NP |  |  |  |  |  |  |  |  |  |  |  |  |  |  |  |  |  | 2 | 1 |  |  |  |  |  |  |  |  |  |
|  | Negat |  |  |  |  |  |  |  |  |  |  |  |  |  |  |  | 2 | 4 | 3 | 2 | 3 | 2 | 2 | 1 | 1 |  | 1 |  |  |
| **Branch** | **VD** |  |  |  |  |  |  |  |  |  |  |  |  |  | **1** |  |  |  |  |  |  |  |  |  |  |  |  |  |  |
| **1B** | SL |  |  |  |  |  |  |  |  |  |  |  |  |  |  |  |  |  |  |  |  |  |  |  |  |  |  |  |  |
|  | NP |  |  |  |  |  |  |  |  |  |  |  |  |  |  |  |  |  | 1 |  | 1,1 |  | 1 |  |  |  |  |  |  |
|  | Negat |  |  |  |  |  |  |  |  |  |  |  | 1 | 4 | 3 | 4 | 3 |  | 1 | 2 | 2 | 2 | 1 | 2 | 1 | 2 | 2 |  |  |
| **Branch** | **VD** |  |  |  |  |  |  |  |  |  |  |  |  |  | **1** |  |  |  |  |  |  |  |  |  |  |  |  |  |  |
| **1C** | SL |  |  |  |  |  |  |  |  |  |  |  |  |  |  |  |  |  |  |  |  |  |  |  |  |  |  |  |  |
|  | NP |  |  |  |  |  |  |  |  |  |  |  |  |  |  |  |  |  | 1 |  | 1 |  |  |  |  |  |  |  |  |
|  | Negat |  |  |  |  |  |  |  |  |  |  |  | 1 | 4 | 3 | 4 | 3 |  |  | 2 | 3 | 2 | 2 | 2 | 1 | 2 | 2 |  |  |
| Branch | VD |  |  |  |  |  |  |  |  |  |  |  |  |  |  |  |  |  |  |  |  |  |  |  |  |  |  |  |  |
| 1AX | SL |  |  |  |  |  |  |  |  |  |  |  |  |  |  |  |  |  |  |  |  |  |  |  |  |  |  |  |  |
|  | NP |  |  |  |  |  |  |  |  |  |  |  |  |  |  |  |  |  |  |  |  |  |  |  |  |  |  |  |  |
|  | Negat |  |  |  |  |  |  |  |  |  |  |  |  |  |  |  |  |  |  |  |  |  |  |  |  |  | 1 | 4 | 3 |
| Branch | VD |  |  |  |  |  |  |  |  |  |  |  |  |  |  |  |  |  |  |  |  |  |  |  |  |  |  |  |  |
| 1AY | SL |  |  |  |  |  |  |  |  |  |  |  |  |  |  |  |  |  |  |  |  |  |  |  |  |  |  |  |  |
|  | NP |  |  |  |  |  |  |  |  |  |  |  |  |  |  |  |  |  |  |  |  |  |  |  |  |  |  |  |  |
|  | Negat |  |  |  |  |  |  |  |  |  |  |  |  |  |  |  |  |  |  |  |  |  |  |  |  |  | 1 | 4 | 3 |
| Branch | VD |  |  |  |  |  |  |  |  |  |  |  |  |  |  |  |  |  |  |  |  |  |  |  |  |  |  |  |  |
| 1AZ | SL |  |  |  |  |  |  |  |  |  |  |  |  |  |  |  |  |  |  |  |  |  |  |  |  |  |  |  |  |
|  | NP |  |  |  |  |  |  |  |  |  |  |  |  |  |  |  |  |  |  |  |  |  |  |  |  |  |  |  |  |
|  | Negat |  |  |  |  |  |  |  |  |  |  |  |  |  |  |  |  |  |  |  |  |  |  |  |  |  | 1 | 4 | 3 |

Sites: Bra, Vrakuna, Bratislava; SkaM= Main inlet to Skalica sewage plant; Coll 1-5, main Skalica collector sewers; Branch 1A-C, main sewers to collector 1; Build 1A1 – 6, sewers from individual buildings leading to Branch 1A; Branch 1AX,-Y,-Z, “illegal” inlets to Branch 1A (cf. Fig.1).

Viruses detected: VD, vaccine derived poliovirus type 2 (red numbers, bold phase), SL poliovirus of any serotype, Sabin-like; NP, non-polio virus, Adenovirus, pink; Rhinovirus, blue, otherwise members of Human enterovirus species A-C (listed in detail in Table S3); Negat, no virus
