# Supplementary material for: Characteristics of an Environmentally Monitored Prolonged Type 2 Vaccine Derived Poliovirus Shedding Episode that Stopped without Intervention
Source: PLoS One. 2013 Jul 31;8(7):e66849. doi: 10.1371/journal.pone.0066849 (PMC3729856; doi:10.1371/journal.pone.0066849)
Supplement: Table S3 — Number of sewage samples positive for different cytopathic viruses. (DOC) [file pone.0066849.s003.doc]

**Table S3.** Number of sewage samples positive for different cytopathic viruses

| Virus | | | Year and number of positive samples | | |
| --- | --- | --- | --- | --- | --- |
| Family | Subgroup/Species | Serotype | 2003 | 2004 | 2005 |
| Picornavirus | HEV-A | CVA16 | 0 | 1 | 1 |
|  | HEV-B | CVB2 | 0 | 0 | 1 |
|  |  | CVB3 | 4 | 0 | 0 |
|  |  | CVB4 | 1 | 2 | 1 |
|  |  | CVB5 | 1 | 8 | 1 |
|  |  | CVB* | 0 | 1 | 0 |
|  |  | E3 | 0 | 25 | 1 |
|  |  | E6 | 3 | 0 | 0 |
|  |  | E11 | 1 | 4 | 0 |
|  |  | E19 | 1 | 0 | 0 |
|  |  | E25 | 1 | 0 | 0 |
|  |  | E30 | 0 | 3 | 0 |
|  | HEV-C | CVA21 | 0 | 0 | 1 |
|  |  | PV1 SL | 0 | 2 | 0 |
|  |  | PV2 SL | 0 | 8 | 0 |
|  |  | PV2 VDPV | 3 | 25 | 7 |
|  | Rhinovirus | NT | 2 | 2 | 0 |
| Adenovirus | NT | NT | 2 | 9 | 1 |
| Annual total | | | 22 | 95 | 15 |

HEV-A/B/C, Human enterovirus A/B/C, respectively.

Sertype column: CVA, coxsackievirus subgroup A; CVB, coxsackievirus subgroup B; *, untyped;

E, echovirus; PV, poliovirus; SL, Sabin-like; VDPV, vaccine derived poliovirus.

NT, not tested
